# *Mycobacterium obuense* Bacteremia in a Patient with Pneumonia

**DOI:** 10.3201/eid2505.180208

**Published:** 2019-05

**Authors:** Bruno Ali López Luis, Paulette Díaz-Lomelí, Livier Patricia Gómez-Albarrán, Areli Martínez-Gamboa, Alfredo Ponce-de-León

**Affiliations:** Instituto Nacional de Ciencias Médicas y Nutrición Salvador Zubirán, Mexico City, Mexico

**Keywords:** bacteremia, pneumonia, immunotherapy, lung, blood cultures, Mycobacteria obuense, pathogenicity, tuberculosis and other mycobacteria, Mexico, bacteria, respiratory infections

## Abstract

*Mycobacterium obuense* is a pigmented, rapidly growing mycobacterium. Because it has been considered nonpathogenic, *M. obuense* is being investigated in clinical trials of cancer immunotherapy and bioremediation. We report a case of bacteremia caused by *M. obuense* in a patient with pneumonia, showing its potential pathogenicity.

Approximately 75 species of rapidly growing mycobacteria (RGM) have been isolated from soil, animals, and water (*1*). The RGM *Mycobacterium obuense*, an environmental pigmented mycobacterium, is mobile and easily adaptable to the environment and possesses oxygenases that enable it to degrade organic compounds and dechlorinate methoxychlor-based insecticides (*2*). Until recently, *M. obuense* has been considered nonpathogenic. We report a case of bacteremia caused by *M. obuense*.

A 29-year-old man from a rural community in Puebla, Mexico, arrived at an emergency department in Mexico City reporting a 2-day history of chest pain, dyspnea, and fever. On physical examination, his heart rate was 94 bpm, blood pressure 175/88 mm Hg, temperature 38.5°C, and peripheral oxygen saturation 70%. Chest auscultation revealed bibasilar fine crackles and signs of pleural effusion. 

The patient was a farmer; had been in close contact with pigs, sheep, and cows; and reported consuming unpasteurized dairy products. He had a history of diabetes mellitus with chronic kidney disease categorized as stage G4 A3 (glomerular filtration rate 16.7 mL/min/1.73 m^2^; proteinuria >2.8 g/d) of the KDIGO classification (Kidney Disease: Improving Global Outcomes, https://kdigo.org) without replacement therapy. He reported taking metformin, amlodipine, furosemide, and iron sulfate.

At admission, laboratory test results included leukocyte count, 11,900 cells/µL with 88.2% neutrophils; C-reactive protein, 250 mg/L; procalcitonin, 17 ng/mL; creatinine, 4.0 mg/dL; and arterial blood gases, pH 7.24, pO_2_ 40.8 mm Hg, pCO_2_ 34.8 mm Hg, lactate 2.9 mmol/L, HCO_3_ 14.6 mmol/L, and sO_2_ 71% on ambient air. Findings of a computed tomography scan of the chest suggested that the patient had a lung infection (Figure).

**Figure:**
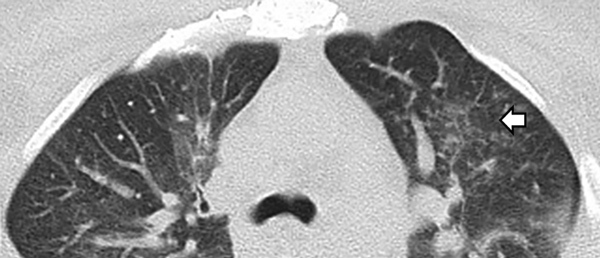
Computed tomography scan of the chest in a patient with *Mycobacterium obuense* pneumonia, Mexico, showing air space infiltration in the left parahilar and a tree bud pattern in the left upper lobe (arrow), as well as bilateral interstitial thickening and ground glass opacities.

The patient began empirical treatment for community-acquired pneumonia with ceftriaxone and clarithromycin. No respiratory samples were obtained because the patient was unable to produce sputum. We performed blood cultures in Aerobic/F medium (Becton Dickinson, https://www.bd.com). After a 7-day incubation period, we detected growth and observed gram-positive bacilli in the gram stain (Appendix Figure, panel A). We made subcultures on sheep blood, chocolate, and Sabouraud agar and performed Kinyoun and Ziehl-Neelsen stains, in which we observed partially acidic alcohol-resistant coccobacilli (Appendix Figure, panels B, C). After 2 weeks of incubation at 35°–37°C, we observed rough, mucoid colonies (Appendix Figure, panels D, E).

We attempted to identify the bacilli by using GenoType Mycobacterium (Hain Lifescience, https://www.hain-lifescience.de) but were unsuccessful because no species-specific probe is available. We performed amplification and sequencing of the 16S rRNA gene (498 bp) and *hsp65* gene (400 bp). Pairwise sequence aligned 100% with 16S rRNA and 99.6% with *hsp65* to the sequences of *M*. *obuense* strain CIP 106803. (GenBank accession no. AF547954.1).

The patient was started on intravenous amikacin, clarithromycin, and moxifloxacin as soon as we notified clinicians of RGM isolated from his blood cultures. We conducted susceptibility testing by broth microdilution, which showed susceptibility to all antimicrobial drugs tested except tobramycin (intermediate susceptibility) (Appendix) (*3*).

The patient completed 2 weeks of intravenous moxifloxacin, amikacin, and clarithromycin and was discharged. He received 4 additional weeks of oral azithromycin and moxifloxacin and experienced complete resolution.

*M. obuense*, first described as a scotochromogenic RGM species isolated primarily from soil >40 years ago in Obu, Japan, is a catalase-positive, peroxidase-negative bacillus that can degrade salicylates, forming a black product (*4*,*5*). In culture, *M. obuense* has 2 morphotypes, smooth and rough variants. In the smooth variants, its cell wall contains long-chain saturated fatty acids that enable it to colonize the environment and are responsible for the pleomorphism observed on the surface of solid agars (*6*). Although this species was later isolated from sputum samples from patients with apparent pulmonary disease, no additional clinical data were reported, and thus, no evidence of pathogenicity was established (*4*,*7*). 

Phylogenetic analysis of *M. obuense* shows close association with *M. chubuense* (81.3% identity) and *M. rufum* (92.2% identity) (*7*). Its genome consists of 5,576,960 bp (of which 133,713 bp are of plasmid origin with 68% GC content) and 800 unique genes, more than related species, such as *M. chubuense.* Although these mycobacteria are considered nonpathogenic, they have homologous genes to mammalian cell entry that encode proteins involved in virulence and cell invasion. In addition, *M. obuense* contains genes involved in antimicrobial resistance, such as *marA*, aminoglycoside-resistance protein kinase, β-lactamases, and monooxygenases, which confer *M. obuense* with intrinsic rifampin resistance (*2*,*7*).

Establishing etiology in this case was challenging because current nucleic acid probe assays cannot identify *M. obuense* correctly. Clinicians should avoid discarding RGM or misclassifying these isolates as colonizers until definitive species identification confirms etiology.

*M. obuense* has been evaluated as an adjuvant immunotherapy in phase I and II trials on patients with melanoma, pancreatic cancer, and colorectal cancer, with promising results. This treatment consists of an intradermal application of a suspension of heat-killed whole cell *M. obuense* (*8*–*10*).

The isolation of *M. obuense* from blood cultures of a patient with community-acquired pneumonia suggests its capacity for virulence and invasiveness in humans. Because *M. obuense* might become an adjuvant in cancer therapy, researchers should ensure implementation of proper, standardized inactivation protocols.

Appendix Results of susceptibility testing and MICs for a variety of antimicrobial drugs. Images of gram stains and agar of *Mycobacterium obuense* from a patient with bacteremic pneumonia.
