# Supplementary material for: Mycobacterium obuense Bacteremia in a Patient with Pneumonia
Source: Emerg Infect Dis. 2019 May;25(5):1015–6. doi: 10.3201/eid2505.180208 (PMC6478194; doi:10.3201/eid2505.180208)
Supplement: Appendix — Results of susceptibility testing and MICs for a variety of antimicrobial drugs. Images of gram stains and agar of Mycobacterium obuense from a patient with bacteremic pneumonia. [file 18-0208-Techapp-s1.pdf]

Article DOI: https://doi.org/10.3201/eid2505.180208

Mycobacterium obuense Bacteremia in a Patient with Pneumonia

Appendix

We conducted susceptibility testing on the bacilli by broth microdilution, which showed MICs to amikacin, 0.25 µg/mL; clarithromycin, $\leq$0.03 µg/mL; ciprofloxacin, 0.03 µg/mL; doxycycline, 0.5 µg/mL; imipenem, 2 µg/mL; linezolid, 0.5 µg/mL; moxifloxacin, $\leq$0.015 µg/mL; tobramycin, 4 µg/mL; and trimethoprim/sulfamethoxazole, 0.25/4.8 µg/mL. According to Clinical Laboratory Standards Institute guidelines (3), all antimicrobial drugs tested were susceptible except tobramycin, which was intermediate.

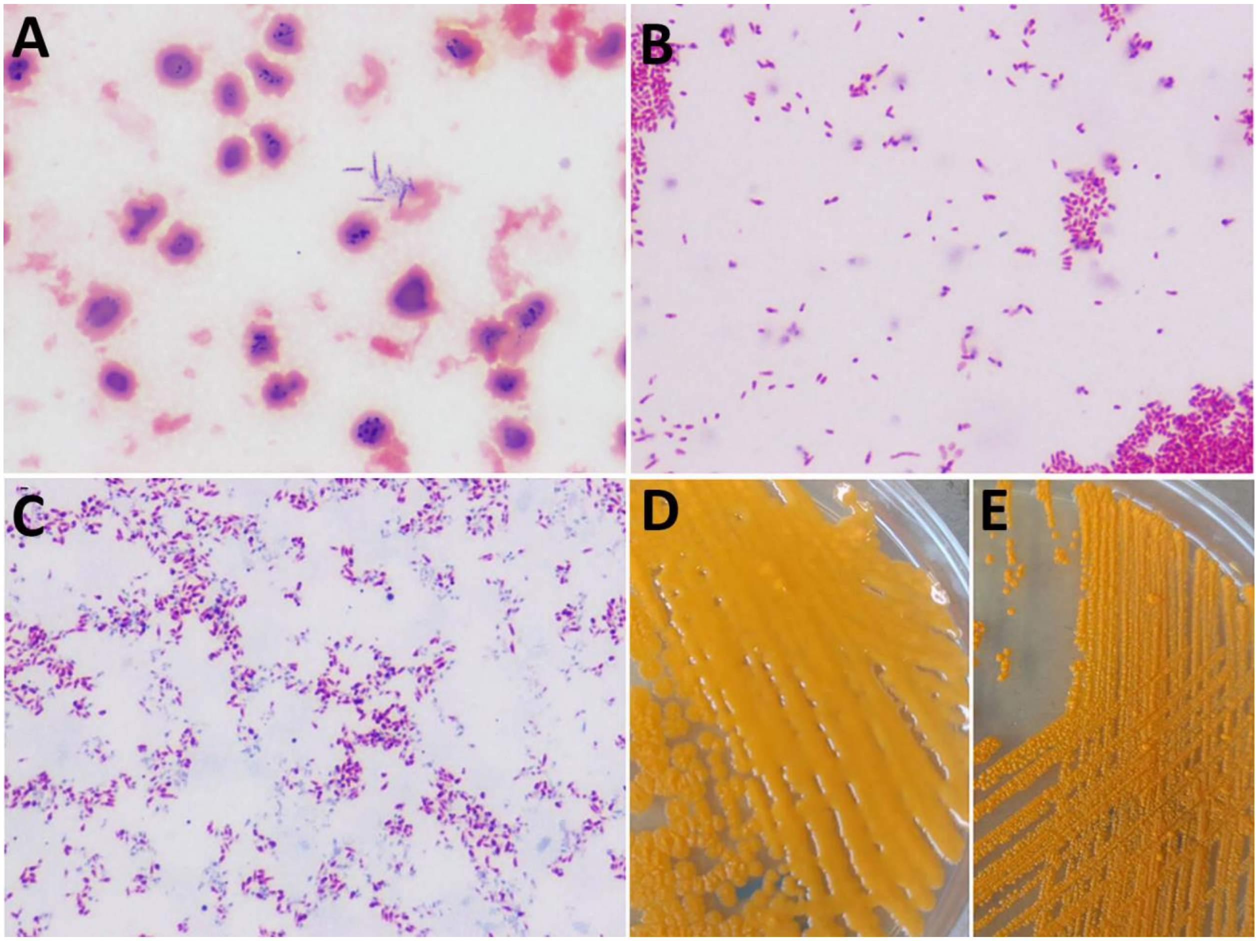

Appendix Figure. A) Gram stain from positive blood culture showing gram-positive bacilli. Original magnification × 100. B) Kinyoun stain from sheep blood agar with red coccobacilli, some in clusters. Original magnification × 100. C) Ziehl Neelsen stain from sheep blood agar showing partially acid-fast stained coccobacilli. Original magnification × 100. D) *Mycobacterium obuense* on Sabouraud agar showing mucoid and smooth colonies with yellow pigmentation. E) *M. obuense* after several passages showing smaller, dry and rough colonies.
